# Virio- and Bacterioplankton Microscale Distributions at the Sediment-Water Interface

**DOI:** 10.1371/journal.pone.0102805

**Published:** 2014-07-24

**Authors:** Lisa M. Dann, James G. Mitchell, Peter G. Speck, Kelly Newton, Thomas Jeffries, James Paterson

**Affiliations:** 1 School of Biological Sciences, Flinders University, Adelaide, South Australia, Australia; 2 School of the Environment, University of Technology Sydney, Sydney, New South Wales, Australia; U. S. Salinity Lab, United States of America

## Abstract

The marine sediment-water interface is an important location for microbially controlled nutrient and gas exchange processes. While microbial distributions on the sediment side of the interface are well established in many locations, the distributions of microbes on the water side of the interface are less well known. Here, we measured that distribution for marine virio- and bacterioplankton with a new two-dimensional technique. Our results revealed higher heterogeneity in sediment-water interface biomass distributions than previously reported with a greater than 45– and 2500-fold change cm^−1^ found within bacterial and viral subpopulations compared to previous maxima of 1.5- and 1.4-fold cm^−1^ in bacteria and viruses in the same environments. The 45-fold and 2500-fold changes were due to patches of elevated and patches of reduced viral and bacterial abundance. The bacterial and viral hotspots were found over single and multiple sample points and the two groups often coincided whilst the coldspots only occurred over single sample points and the bacterial and viral abundances showed no correlation. The total mean abundances of viruses strongly correlated with bacteria (r = 0.90, p<0.0001, n = 12) for all three microplates (n = 1350). Spatial autocorrelation analysis via Moran’s I and Geary’s C revealed non-random distributions in bacterial subpopulations and random distributions in viral subpopulations. The variable distributions of viral and bacterial abundance over centimetre-scale distances suggest that competition and the likelihood of viral infection are higher in the small volumes important for individual cell encounters than bulk measurements indicate. We conclude that large scale measurements are not an accurate measurement of the conditions under which microbial dynamics exist. The high variability we report indicates that few microbes experience the ‘average’ concentrations that are frequently measured.

## Introduction

Marine ecosystems are dependent on the microbial loop to carry out critical marine biogeochemical processes such as carbon and nitrogen fixation [1]–[8]. These marine biogeochemical processes often occur at or near cell surfaces with interactions occurring over micrometres to centimetres [9]–[17]. However, marine microbe distributions are commonly measured in large volumes and then that value is extrapolated over larger areas or volumes in what is sometimes termed a ‘mean field’ approach [12], [18]–[24].

There is growing awareness of deficiencies in this mean field approach for productivity, biomass and process estimates [12], [25]–[28]. Studies on patch dynamics in terrestrial and aquatic ecosystems show most organisms have heterogeneous spatial distributions. This patchiness would be missed if the sampling resolution used was not appropriate for the size of the organism studied [29]–[32]. For instance, Brentnall *et al.* [33] found that the mean field production of planktonic communities greatly underestimated actual production due to small scale patchiness. The little work on picoplankton distributions indicates that there is microscale spatial heterogeneity of bacteria and viruses, and that ‘hotspots’ and ‘coldspots’ are common, with bacterial and viral abundance changing 5-fold and 1.4-fold cm^−1^ [15], [34].

Hotspots are areas of high microbial abundance which are believed to result from interactions between microbes and organic matter [9], [12], [13], [15], [35]. These viral and bacterial hotspots were often found to be due to bacterial response to nutrient patches, as marine bacteria have been found to accumulate around areas of high nutrient concentration via chemotaxis [9], [31], [36]–[40], This aggregation of bacteria then results in bacterial hotspots that are often followed by increased viral production via lysis due to close host proximity [15]. In contrast, coldspots are areas of low microbial abundance that are believed to be a result of lysis or grazing events in the case of lowered bacterial abundance and grazing or attachment in the case of lowered viral abundance [17].

The presence of these hotspots and coldspots are the units of microscale patchiness. The lower size limit of patches is controlled by the Batchelor scale, which is the smallest scale that nutrient gradients can occur before being dispersed by diffusion [41], [42]. Chemotactic bacteria can exploit ephemeral nutrient gradients above the Batchelor scale. This exploitation leads to hotspots in bacterial abundance [37], [38]. Patchiness in bacterial and viral distributions are also dependent on the season and time of sampling [13], [39]. For instance, previous studies have shown bacterial abundance and activity were higher in the late afternoon [13]. This study ensured sampling was taken at the same time of day and within the same season to avoid confounding from these low frequency signals.

Although hotspots are a ubiquitous feature of microbial distributions they are often vaguely defined in studies, primarily being classified as patches of high microbial abundance that ‘exceed’ or are ‘elevated above’ background variation across one or two sampling points [12], [13], [43]. The methods for determining background are not given and the amount of elevation is rarely stated and differs considerably between studies making comparative analysis difficult. For instance, in the case of Seymour *et al.* [12] the data shown for hotspots suggests that elevation above background is less than one order of magnitude whereas in Seymour *et al.* [43] a conservative value of ≥4-fold higher is considered as a hotspot. In addition to this, coldspots have also lacked a definitive characterisation despite being observed in the microscale distributions of microbial communities previously [12], [15], [17].

As well as hotspots and coldspots, surface gradients are a common pattern seen in microbial distributions, and are seen when bacterial and viral abundance is highest at an interface and then dissipates into the water column as the distance from the interface increases. These surface gradients arise from the sinking of organic matter and its incorporation into the benthos followed by degradation and transformation by microbes leading to high nutrient concentrations and hence high bacterial and viral concentrations directly above the sediment-water interface [35]. The microscale distributions of microbial communities on the sediment side of the interface are well established, whilst the distributions of microbes on the water side of the interface are less well known and where this has been investigated the focus has been largely on bacteria [12], [15], [34]. However, of the studies analysing the water side, surface gradients have been seen in the distributions of bacterial and viral subpopulations above coral colonies [14], in the water column above an anchialine sinkhole [44] and in coastal [12] and estuary environments [15], [35]. Viral studies show the greatest level of heterogeneity is found 1–2 cm above the sediment-water interface with abundance at St Kilda mangroves in South Australia varying by more than 2-fold, over a 15 cm sampling distance, about 1.13-fold cm^−1^, with the highest concentrations found 1.5 cm from the sediment-water interface [15], [35]. Similar patterns were seen in the vertical profiles in Seymour *et al.* [35] which showed high viral and bacterial abundance closest to the sediment-water interface with approximate increases of 1.4- and 1.5-fold cm^−1^ in total virus and total bacteria. However, due to one-dimensional sampling and lack of replication, determining whether these changes in abundance were single or multiple point maxima or surface gradients was unknown.

The null hypothesis tested in this study was that patchiness has no effect on mean biomass estimates and therefore the mean field approach is an accurate estimate of biomass. To test this hypothesis, two-dimensional sampling with extensive replication was used which builds upon previous work by Seuront *et al.* [45], Seymour *et al.* [13]–[15], [35], [43] and Waters *et al.* [17]. Here, a new sampler with 9 mm resolution provided replication via the collection of 8 vertical profiles per sampler or 24 profiles per environment, which is much larger than the previous maxima of 5 single vertical profiles per environment [35]. This replication allowed a detailed picture of microbial distributions immediately above the sediment water interface. The value of this design is that it shows how much the ‘mean field’ approach underestimates absolute concentration and misses the resources, competition and viral exposure gradients that microbes experience.

## Materials and Methods

### Study site

Samples were collected from Saint Kilda mangroves and Port Noarlunga in South Australia. These environments were chosen to build on previous work using these sites [12], [13], [15], [35]. The site at Port Noarlunga is located near the mouth of the Onkaparinga River and is bordered by sand dunes covered in remnant vegetation. Previous studies have described the water at Port Noarlunga as oligotrophic [12]. Samples were collected from Port Noarlunga (35°16′S, 138°47′E) on the 12^th^ of July 2011 at 11∶20 am; the temperature of the water was 12.3°C with a pH of 9.4, a dissolved O_2_ level of 14.3 ppm and a conductivity value of 40.7 ppt.

The St Kilda site was characterised by hypersaline lagoons less than 1 m deep in dense mangrove forests. Previous studies have shown St Kilda mangroves host a microbial community that is characterised by highly productive microbial mats and sulphur-oxidising bacteria [12], [13], [35], [46]. Samples were collected from St Kilda (34°74′S, 138°54′E) on the 6^th^ of July 2011 at 10∶00 am; the temperature of the water was 11.5°C with a pH of 8.5, a dissolved O_2_ level of 14.3 ppm and a conductivity value of 41.5 ppt.

Environmental conditions data was collected using a Hydrolab Datasonde 4a sensor. Permission to access the sampling site from the St Kilda mangrove trail site was provided by the National Parks and Wildlife Rangers of the City of Salisbury Council. Specific permission to access the Noarlunga sampling site was not required. The field studies did not involve endangered or protected species.

### Sampling device (and supplementary methods)

Microplate triplicates were used to measure the microscale spatial distribution of marine viral and bacterial populations. Corning Costar TC-treated cell culture cluster microplates were used. They were 8 cm×12.3 cm, nonpyrogenic, polystyrene and sterile, consisting of 96 flat bottom 8 mm×8 mm wells which held 360 µl per well. The centre to centre distance between wells was 9 mm and the well perimeters were 7.2 cm at the ends and 10.8 cm on the sides resulting in a collection area of about 77.8 cm^2^. This allowed the collection of 8 vertical profiles per microplate, which each consisted of 12 sampling points (Figure S1). Microplate triplicates gave 24 vertical profiles. The sampler was placed vertically against the respective surface and removed in a vertical motion with a 16 cm×16 cm glass plate used as a cover to minimise mixing and turbulence when collecting samples.

### Sample collection and preservation

All samples were transferred into 2 ml cryovials containing 4 µl of glutaraldehyde (0.5% final concentration) using a pipette and stored at 4°C in the dark for 15 minutes. Samples were then snap frozen in liquid nitrogen and stored in a −80°C laboratory freezer [47]. Flow cytometric analysis was performed within three weeks to avoid deterioration [47].

### FCM sample preparation and analysis

Many previous studies have used flow cytometry for the enumeration of bacteria and viruses due to its rapidity and accuracy [48], [49]. Here we use the same flow cytometry technique as previous studies [12]–[15], [34], [35], [43] and built on these studies by adding extensive replication. Briefly, samples were defrosted and prepared for flow cytometric viral and bacterial enumeration by diluting each sample 1∶10 with Tris-EDTA buffer (pH 8.0, 0.2 µm filtered, 10 mM Tris, 1 mM EDTA) and staining with a nucleic acid-specific dye, SYBR Green I (1∶500 dilution commercial stock; Molecular Probes). Samples were then incubated at 80°C in the dark for 10 minutes to optimise viral counts [35], [47]–[51]. Reference beads (1 µm diameter, Molecular Probes) were added as an internal concentration and size standard with a final concentration of approximately 10^5^ beads ml^−1^ in each sample. Measured flow cytometry parameters were normalised to the fluorescence and concentration of these beads [4], [47]–[51].

Flow cytometry was conducted on a FACSCanto II flow cytometer (BD) equipped with a blue (488 nm, 20 mW, air-cooled), red (633 nm, 17 mW) and violet (405 nm, 30 mW) laser. Forward-angle light scatter (FSC), right-angle light scatter (SSC) and green fluorescence (SYBR I) were acquired for each sample and a phosphate-buffered saline (PBS) solution was used as a sheath fluid. The flow cytometer settings were normalised to fluorescence and bead concentration [35], [50]. Each sample was run for two minutes at a medium flow rate setting for Noarlunga samples and a low flow rate setting for St Kilda samples due to more suspended particulate matter being present in St Kilda samples. In each flow cytometry session, triplicate blank control samples were analysed which consisted of 500 µl of 0.2 µl filtered Tris-EDTA buffer stained with 12.5 µl of SYBR Green I to eliminate any background noise that may have been created during the sample preparation or from flow cytometer artifacts.

### Sample processing

The resulting cytograms, density plots and histograms acquired from flow cytometry were exported as listmode files and analysed using Win Midi 2.9 (Joseph Trotter) to enumerate the bacterial and viral populations present [35]. Viral and bacterial populations were discriminated via side-scatter (SSC), which indicates cell size; and SYBR Green fluorescence, which is indicative of nucleic acid content [47], [48], [51].

### Data analysis and representation

For this study, rank abundance graphs were used to discriminate between hotspots, coldspots and background values. The background was determined according to Weibe [52] where the median value of the dataset is used as the background due to inclusion or exclusion of hotspots or coldspots not overly affecting the values obtained.

Surfer 10 (Golden Software, Inc.) was used to create two dimensional contour plots of the spatial distribution of viral and bacterial populations. A minimum contour interval value of at least 1000 was chosen when constructing plots due to this being larger than the maximum FACSCanto II flow cytometer error observed. This error refers to the background noise seen within triplicate blank control samples during flow cytometric analysis. This value is conservative as the maximum machine error was less than 36 events µl^−1^.

Correlations were determined via Pearson’s coefficient with the α of 0.05 being reduced by sequential Bonferroni [53]. Spatial autocorrelation statistics were used to determine whether correlations were present two-dimensionally and multi-directionally between sample points that were proximate and had similar values. From this, values were derived that illustrated the spatial autocorrelation present in the dataset. The two most common geospatial statistical tests are Moran’s I and Geary’s C statistics and both of these were employed as they allow statistical testing on smaller datasets [17].

#### Moran’s I Statistic

Moran’s *I* spatial autocorrelation statistics test within CrimeStat 3.3 (courtesy of Ned, Levine software) was used to determine the level of randomness within each population. The equation is as follows:
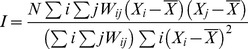
Where N is the sample number, X_i_ and X_j_ represent the variable values at a specific locations, i and j (where i 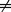 j), 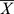 is the mean of the variable and W_ij_ is the weight applied to the i and j comparison. A weighted Moran’s I test was used which gave a weight value of 1 to sample points adjacent and a weight value of 0 to sample points not adjacent.

The Moran’s I statistical test [54] is a global statistical test used to determine spatial autocorrelation within a set of values. It is multi-directional, being able to use vertical, horizontal and diagonal directional analysis to consider all position correlations. The Moran’s I value ranges from +1 to −1, with +1 indicating perfect clustering where high values are proximate and low values are proximate, −1 indicating perfect dispersion where high values are found far apart and low values are found far apart and zero being indicative of a random distribution. Critical cut-off values for most statistical tests are dependent on the scale and values used and are often well established through previous literature using the same or similar types of studies. However, in the instance of Moran’s I values for small-scale spatial studies of bacteria and viruses there is only 1 study to compare which I values are indicative of significance. Waters et al. [17] looked at phytoplankton distributions on a 2 cm and 4 cm scale. From looking at the I values obtained in the Waters et al. [17] study, it appears I values between 0.08 and 0.18 indicate a considerable level of clustering, as opposed to a random distribution, which is far less than the general clustering I value of +1.

#### Moran Correlograms

Moran correlograms were constructed using the Moran’s I statistic, which is applied to pairs of sample values separated by a lag distance, which in this case was the distance between each sampling well (0.9 cm). Geostatistical analysis requires each lag distance to have ≥30 pairs of sample points to enable statistical reliability; therefore sample intervals with less than this were not included in the correlograms [17], [55]. The standard error obtained via Moran’s I analysis was used to determine if correlograms and lag distance values were significant. Output values were used to determine whether spatial autocorrelation or independence was present at each distance interval from the sediment-water interface.

#### Geary’s C Statistic

Geary’s C statistics [56] test within CrimeStat 3.3 (courtesy of the Ned, Levine software) is a global statistic that can be used in conjunction with Moran’s I as it is more sensitive to local clustering and is able to identify deterministic patterning of extreme values and non-spatially related chance phenomena within the dataset, which cannot be identified using Moran’s I alone. Geary’s C is similar to Moran’s I, however it calculates the spatial autocorrelation of a dataset by the deviation in intensity of each sample value’s location compared to one another, whereas in Moran’s I spatial autocorrelation is calculated by the cross-product of the deviations from the mean within the dataset. The equation is as follows:
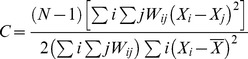



All terms are the same as Moran’s I. For Geary’s C, the values range from 0 to approximately 2, however there is no definitive upper limit [57]. A value of 1 indicates spatial independence, whilst values <1 indicate positive spatial autocorrelation and values >1 indicate negative spatial autocorrelation. Therefore this statistical test is inversely related to the Moran’s I test [58]. The Moran’s I and Geary’s C can be used in conjunction to identify deterministic patterning of extreme values [57].

## Results

### FCM analysis

**Figure 1:**
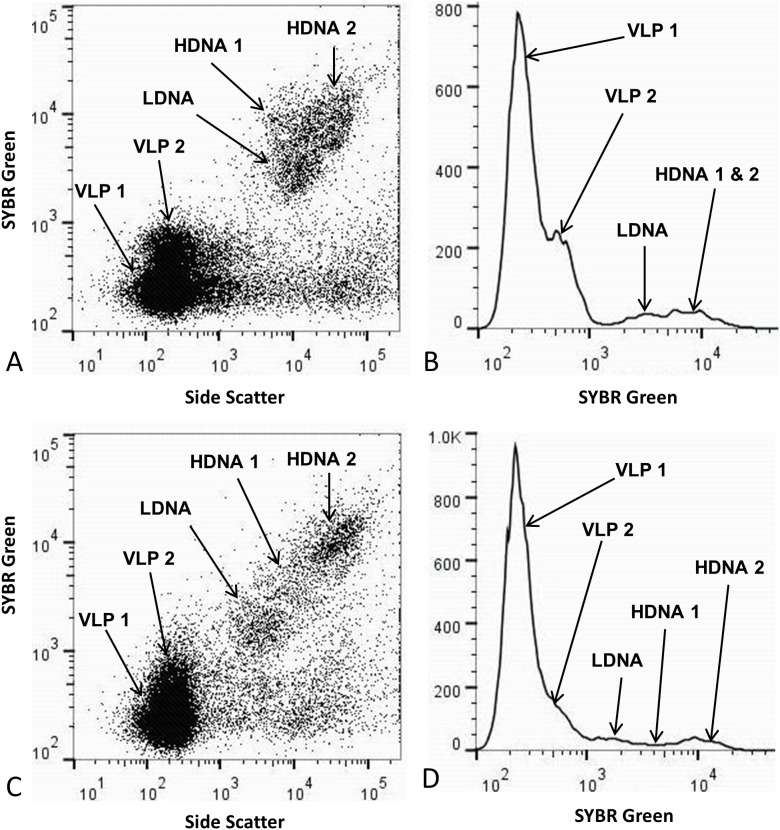
Identification of bacterial and viral subpopulations via flow cytometry. Flow cytometric cytograms of side-scatter light versus green fluorescence (SYBR Green) and histograms of green fluorescence (SYBR Green). **Noarlunga:** **A** cytogram **B** histogram; **St Kilda:** **C** cytogram **D** histogram showing two distinct viral populations (VLP 1 and VLP 2) and three distinct bacterial populations (LDNA, HDNA 1 and HDNA 2).

Flow cytometric analysis revealed three distinct bacterial subpopulations and two distinct viral subpopulations at both sites which were comparable to those identified previously in marine systems [12], [15], [48]. Subpopulation discrimination was based on the presence of aggregated regions on biparametric cytograms of side scatter and SYBR green fluorescence and discrete peaks in monoparametric histograms of SYBR green fluorescence (Figure. 1A–D). Bacterial subpopulations were differentiated and separated into high-DNA (HDNA) and low-DNA (LDNA) groups (Figure. 1A–D). The HDNA 1 bacterial population is the intermediate bacterial population that contains bacteria with a size and nucleic acid content that is intermediate to the LDNA and HDNA 2 populations. The two viral subpopulations were identified and separated into two virus-like particle subpopulations (VLP 1 and VLP 2).

At Noarlunga, the total mean abundance of the VLP 1 subpopulation was 3.7×10^6^ cells ml^−1^ (95%CI = 0.3×10^6^ cells ml^−1^, n = 269), whereas it was 1.3×10^6^ cells ml^−1^ for the VLP 2 subpopulation (95%CI = 0.1×10^6^ cells ml^−1^, n = 269) (Figure. 1A and 1B). Hence, the total mean abundance of VLP 2 was 2.8-fold less than the total mean abundance of VLP 1. At St Kilda, the total mean abundance of VLP 2 was also less than VLP 1, but to a greater extent as the total mean abundance of VLP 1 was 75×10^5^ cells ml^−1^ (95%CI = 5.1×10^5^ cells ml^−1^, n = 209) compared to 6.1×10^5^ cells ml^−1^ (95%CI = 0.45×10^5^ cells ml^−1^, n = 209) for VLP 2 (Figure. 1C and 1D). Hence, the total mean abundance of VLP 2 was 12.3-fold less than the total mean abundance of VLP 1.

For the bacteria, the LDNA subpopulation at Noarlunga was less abundant than the HDNA 1 and the HDNA 2 subpopulations. The total mean abundance for LDNA was 0.4×10^5^ cells ml^−1^ (95%CI = 0.03×10^5^ cells ml^−1^, n = 269) compared to total mean abundances of 3.1×10^5^ cells ml^−1^ (95%CI = 0.2×10^5^ cells ml^−1^, n = 269) for HDNA 1 and 2.7×10^5^ cells ml^−1^ (95%CI = 0.2×10^5^ cells ml^−1^, n = 269) for HDNA 2 (Figure. 1A and 1B). Hence, the total mean abundance of LDNA was up to 7-fold less than the total mean abundance of HDNA 1 and HDNA 2. Whereas at St Kilda, HDNA 1 was the least abundant bacterial subpopulation (Figure. 1C and 1D) with total mean abundances of 3.0×10^5^ cells ml^−1^ (95%CI = 0.32×10^5^ cells ml^−1^, n = 209) for LDNA, 2.1×10^5^ cells ml^−1^ (95%CI = 0.47×10^5^ cells ml^−1^, n = 209) for HDNA 1 and 5.9×10^5^ cells ml^−1^ (95%CI = 1.7×10^5^ cells ml^−1^, n = 209) for HDNA 2. Hence, the total mean abundance of HDNA 1 was 2-fold less than the total mean abundances of LDNA and HDNA 2 (Table S1 and S2).

### Vertical profiles

**Figure 2:**
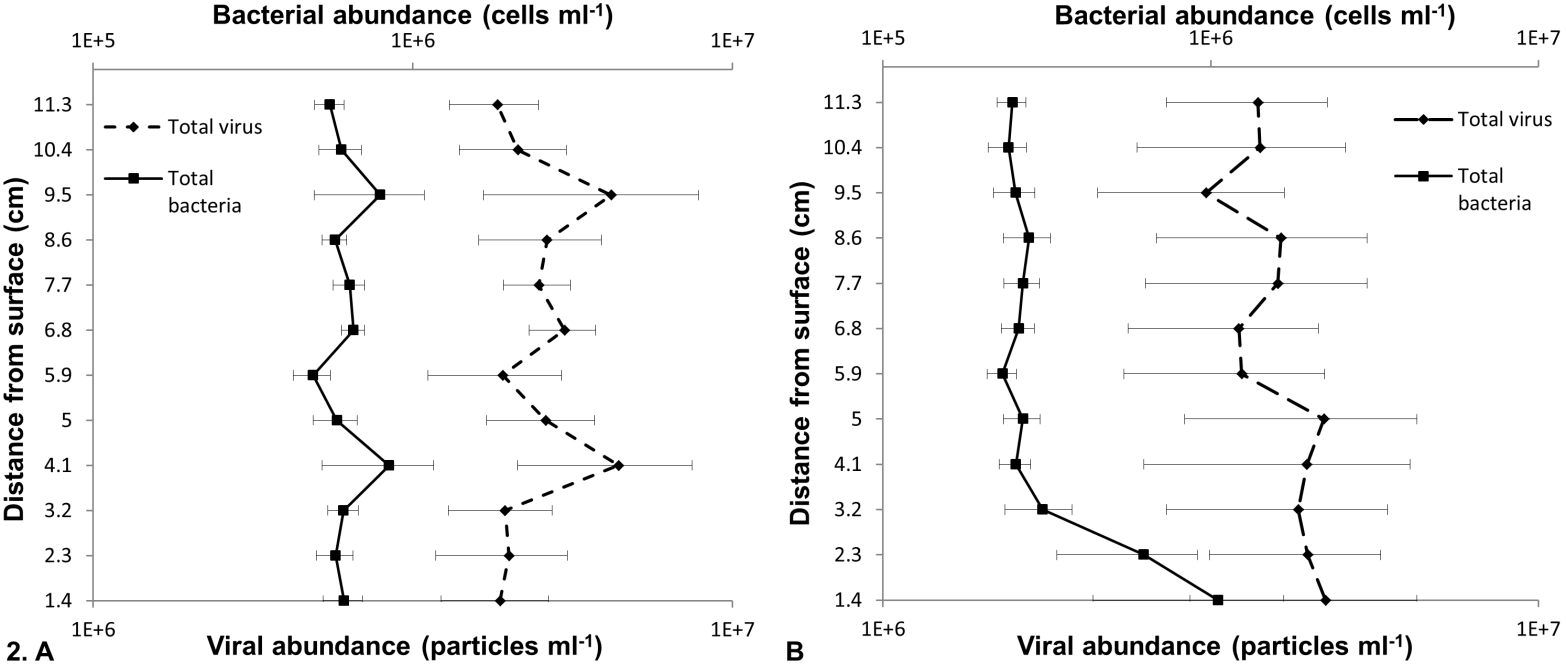
Comparison of total mean bacterial and viral populations at Noarlunga via vertical depth profiles. Total mean bacterial and total mean viral population abundance within all three microplates. **A** Noarlunga, (n = 1350). **B** St Kilda, (n = 1045). Error bars represent the 95% confidence intervals obtained from all three replicates (n = 12).

**Figure 3:**
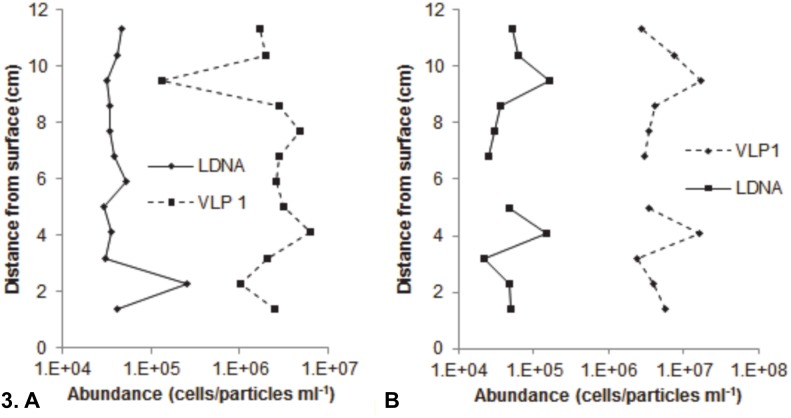
Single vertical profile of VLP 1 and LDNA populations at Noarlunga. **A** Microplate three showing little to no association. **B** Microplate one showing association. Gap in profile indicates a missing data point.

At Noarlunga, the mean of 24 vertical profiles had an r value of 0.9 (p<0.0001, n = 12) and showed almost identical patterns between the total mean bacterial and total mean viral population (Figure. 2A), as well as the total mean LDNA, HDNA 1 and HDNA 2 subpopulations (Figure. S2A), the total mean VLP 1 and VLP 2 subpopulations (Figure. S2B) and the mean vertical profile of VLP 1 and LDNA from microplate 1, which all had an r value of 0.99 (p<0.0001, n = 12) (Figure. S3). In addition, 145 of the 240 possible bacterial and viral single vertical profile subpopulation correlations were significantly correlated with a p-value generally less than 0.0001, such as the single vertical profile of VLP 1 and LDNA from microplate 1 which had an r value of 0.98 (p<0.0001, n = 12) (Figure. 3B). However, 95 of the single vertical profiles were not significantly correlated, such as the single vertical profile of VLP 1 and LDNA from microplate 3 which had an r value of −0.33 (p = 0.12, n = 12) (Figure. 3A). At St Kilda, the mean of 24 vertical profiles was not significantly correlated (Figure. 2B). However, the total mean LDNA, HDNA 1 and HDNA 2 populations were correlated (r = 0.98, p<0.0001, n  = 12), as well as the total VLP 1 and total VLP 2 populations (r = 0.64, p = 0.02, n = 12). Of the 240 possible bacterial and viral single vertical profile subpopulation correlations, only 64 were significantly correlated with a p-value generally less than 0.0001, whilst 176 were not correlated.

### Determination of hotspots and coldspots

**Figure 4:**
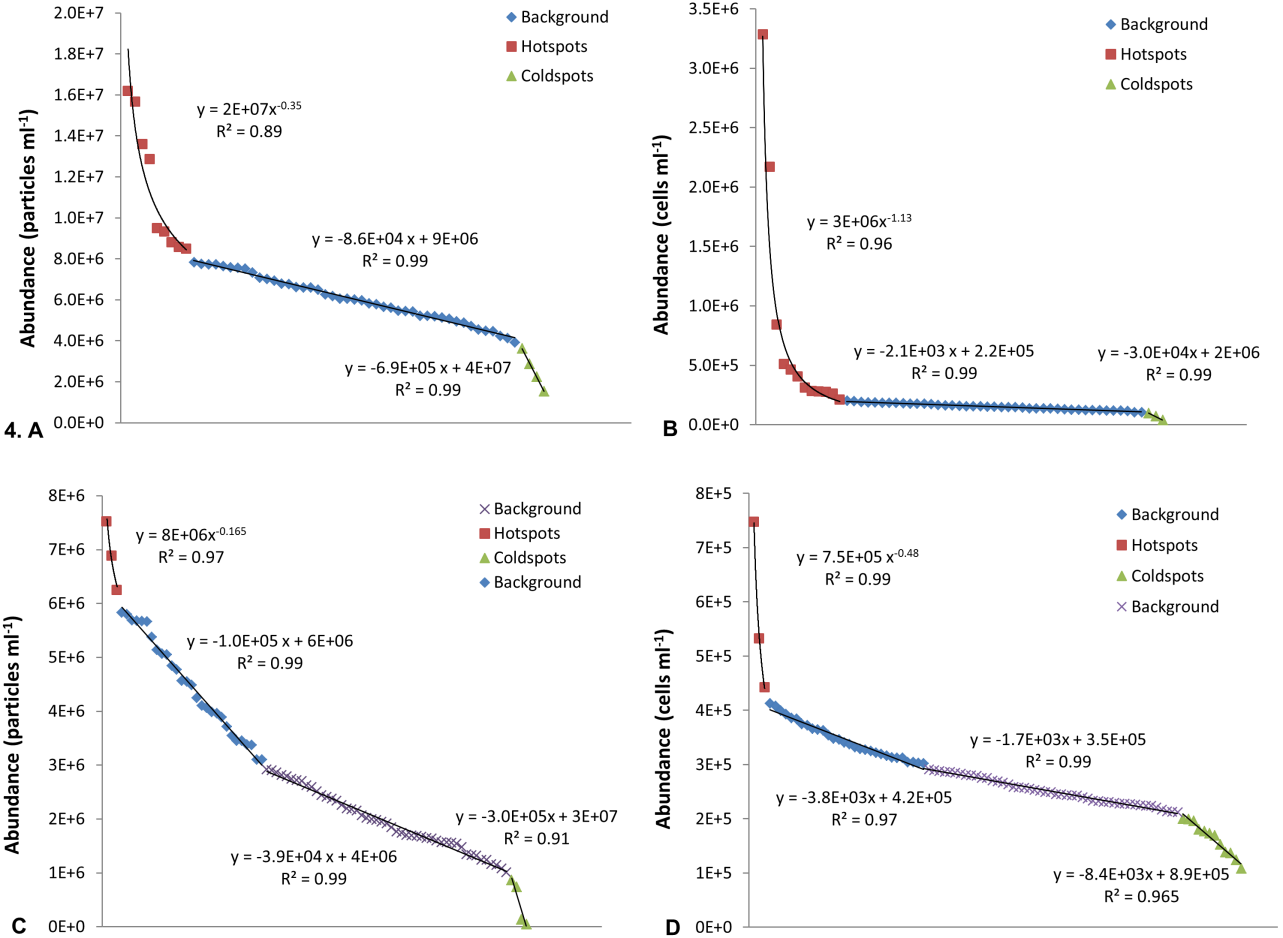
Rank abundance graphs used to differentiate hotspot and coldspot values from background values. One linear trend within the background values were characteristic of St Kilda whilst two linear trends were primarily seen at Noarlunga. **St Kilda: A** VLP 1, microplate 2. **B** HDNA 1, microplate 2. **Noarlunga: C** VLP 1, microplate 3. **D** HDNA 2, microplate 2.

To determine the presence of hotspots and coldspots in the viral and bacterial distributions, rank abundance graphs were created (Figure. 4A–D). In some instances only one linear trend was seen within the background values and in others there were breaks in the fits that indicated two or three separate linear trends. St Kilda showed primarily one linear trend, for instance in the VLP 1 and HDNA 1 subpopulations (Figure. 4A–B) whilst Noarlunga showed primarily two linear trends within their background values, for instance in the VLP 1 and HDNA 2 subpopulations (Figure. 4C–D). The hotspots were identified as the sample points that exceeded this linear fit (Figure. 4A–D). Exponent values for these hotspots ranged from −0.20 to −1.1 for the bacteria and −0.14 to −0.63 for the viruses at Noarlunga, whilst exponent values at St Kilda ranged from −0.27 to −1.4 for the bacteria and −0.23 to −0.66 for the viruses (Figure. 4A–D). Lastly, the coldspot sample values were identified as sample points that fit a linear trend but exhibited steeper slopes than what were seen in the background values due to large differences between adjacent sample points (Figure. 4A–D).

### Two dimensional distributions

**Figure 5:**
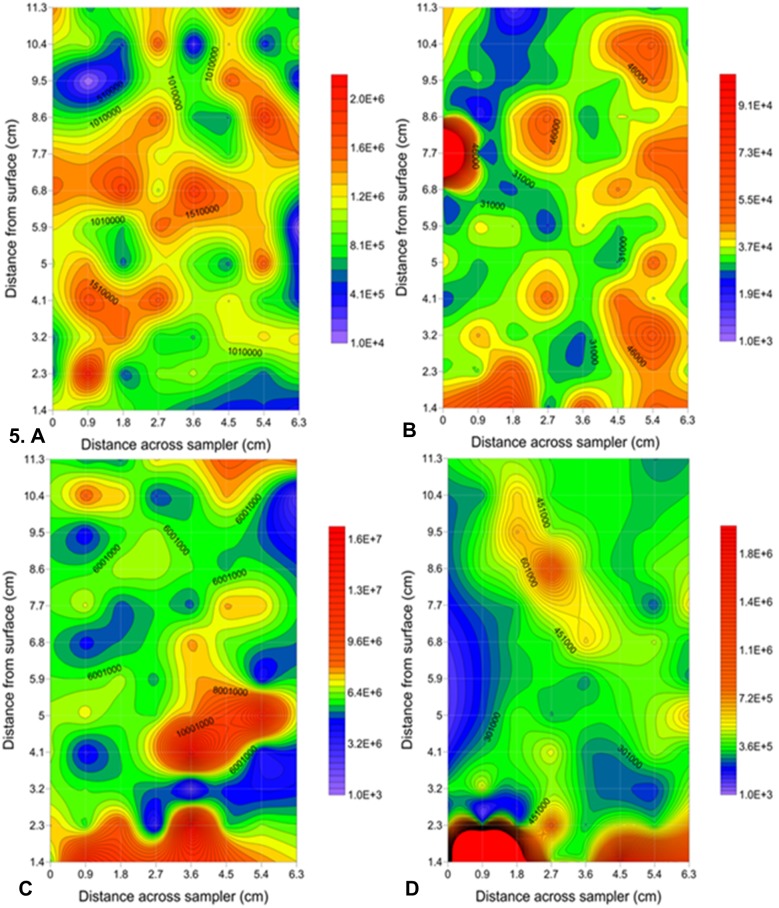
Two-dimensional contour plots showing the highest change in heterogeneity due to the presence of hotspots and coldspots within bacterial and viral subpopulations. Hotspots and coldspots seen across a distance of 6.3×11.3 cm using Surfer 10 (Golden Software, Inc.). Noarlunga: A VLP 2 showing a 2585-fold change in abundance over 0.9 cm. B LDNA showing a 12.9-fold change in abundance over 0.9 cm. St Kilda: C VLP 1 showing a maximum 10.52-fold change in heterogeneity seen over 0.9 cm. D HDNA 2 showing a maximum 45.2-fold change in heterogeneity seen over 0.9 cm. There were a range of heterogeneities over 0.9 cm (Fig. S4) indicating a variety of intensities for hotspots and coldspots. Abundance levels are indicated by a colour intensity scale in units of cells/particles ml^−1^. Solid red circles indicate areas of abundance higher than the maximum contour level selected. A minimum contour interval value of at least 1000 was chosen based on maximum machine error. The faint gridlines show sample interval.

Two-dimensional contour plots of both sampling sites showed heterogeneous distributions with patches or hotspots of high abundance that occurred over single or multiple sample points as well as gaps or coldspots of low abundance that occurred only over single sample points (Figure. 5A–D).

At Noarlunga, high levels of heterogeneity were seen, with the VLP 2 subpopulation showing the highest level of heterogeneity of the two viral subpopulations. It was due to the presence of coldspots in the VLP 2 subpopulation that resulted in a 2585-fold change in abundance over 0.9 cm, with a coldspot of 6.64×10^2^ particles ml^−1^ below a background of 1.07×10^6^ particles ml^−1^ (Figure. 5A). For the bacterial subpopulations, the highest level of heterogeneity was seen in the LDNA subpopulation with a maximum hotspot of 2.59×10^5^ cells ml^−1^ above a background of 3.47×10^4^ cells ml^−1^ resulting in a 12.9-fold change in abundance over 0.9 cm (Figure. 5B).

At St Kilda, the highest viral heterogeneity was present in the VLP 1 subpopulation which had a maximum hotspot of 1.62×10^7^ particles ml^−1^ above a background of 6.12×10^6^ cells ml^−1^ which resulted in a 10.52-fold change in heterogeneity seen over 0.9 cm (Figure. 5C). For the bacterial subpopulations at St Kilda a 45.2-fold change in heterogeneity was seen in the HDNA 2 subpopulation due a maximum hotspot of 1.45×10^7^ cells ml^−1^ above a background of 3.7×10^5^ cells ml^−1^ (Figure. 5D). There were a range of heterogeneities over 0.9 cm (Figure. S4) indicating a variety of intensities for hotspots and coldspots. Representative adjacent orthogonal gradient distributions as rank abundances show the distribution of abundance values for selected bacterial and viral populations at St Kilda and Noarlunga (Figure. S4).

### Spatial autocorrelation

#### Moran’s I

At Noarlunga, the Moran’s I values for all of the bacterial subpopulations were non-significant. Whereas at St Kilda, significant Moran’s I values were found in 33% of the bacterial subpopulations; the LDNA subpopulation of microplate 2 and 3 and the HDNA 2 subpopulation and total bacteria of microplate 2. These significant Moran’s I values ranged from 0.039–0.070, with all the non-significant Moran’s I values being below these values (≤0.036). For these bacterial subpopulations z scores under the assumption of randomisation were much larger than the cut-off point of +1.96, with z scores ranging from 2.5 to 3.8. All the viral subpopulations at Noarlunga and St Kilda had non-significant Moran’s I values.

**Figure 6:**
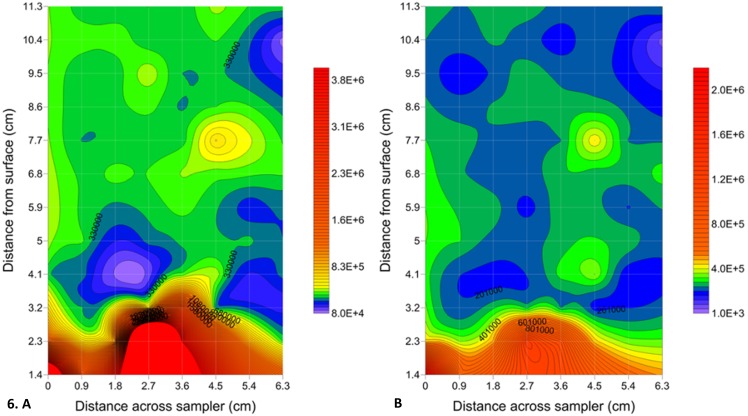
Two-dimensional contour plots showing surface gradients within bacterial subpopulations at St Kilda. Surface gradients seen across a distance of 6.3×11.3 cm using Surfer 10 (Golden Software, Inc.). Microbial abundance levels are indicated by a colour intensity scale in units of cells ml^−1^. **A** HDNA 2, microplate 2. **B** LDNA, microplate 2. Solid red circles indicate areas of abundance higher than the maximum contour level selected. A minimum contour interval value of at least 1000 was chosen based on maximum machine error. The faint gridlines show sample interval.

All of the significant Moran’s I values for the bacterial subpopulations were positive (Table S3 and S4). This trend is evident in the 2 dimensional contour plots of HDNA 2 and LDNA at St Kilda which show the presence of sediment-water interface surface gradients (Figure 6A–B). Statistical significance was not found for negative Moran’s *I* values (Table S3 and S4). In geographic data, statistically significant positive values are more common than statistically significant negative values.

#### Moran correlograms

**Figure 7:**
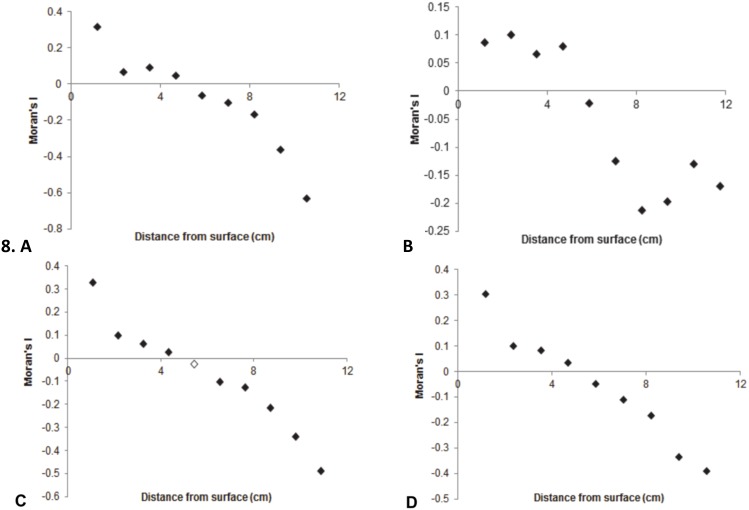
Significant Moran correlograms of non-randomly distributed bacterial subpopulations at St Kilda. **A** LDNA, microplate 2. **B** LDNA, microplate 3. **C** HDNA 2, microplate 2. **D** Total bacteria, microplate 2. Filled and unfilled data points indicate significant and non-significant Moran’s I values (p≤0.01). Only sample points with ≥30 pairs of values were included.

Moran correlograms for the bacterial subpopulations that had significant Moran’s I values showed a general trend of positive to negative spatial autocorrelation as the distance from the sediment-water interface increased. On closer inspection, the correlograms for these bacterial subpopulations showed positive spatial correlation in the sample points located within 5.9 cm of the sediment-water interface and spatial independence as this distance from the sediment-water interface increased at >5.9 cm (Figure. 7A–D). This is indicative of surface gradients, which are shown in figures 6A–B. Moran correlograms for the LDNA, HDNA 1 and total bacteria in microplate 1, and HDNA 1 in microplate 2 at St Kilda also followed this trend despite having non-significant Moran’s I values. Geary’s C statistical testing was used to further analyse these subpopulations.

At Noarlunga and St Kilda, all of the viral subpopulation correlograms were non-significant and showed no general trend, with their distributions alternating between positive and negative spatial autocorrelation throughout the sampling distance indicating the presence of hotspots and coldspots.

#### Geary’s C

At Noarlunga, significant Geary’s C values were obtained from all of the bacterial subpopulations in microplate 1, whilst all of the bacterial subpopulations in microplate 2 and 3 were non-significant. Geary’s C values were between 0.87 and 1.02; however, significance was only seen in C values of 0.87 (Table S3 and S4).

In contrast, at St Kilda significant Geary’s C values were seen in 75% of the bacterial subpopulations; all of the subpopulations in microplate 1 and 2, as well as LDNA within microplate 3. Significant C values were between 0.73 and 0.92 with C values between 0.96 and 0.99 showing non-significance (Table S3 and S4).

At Noarlunga, significant Geary’s C values were found in the total virus and VLP 2 subpopulation in microplate 1 whilst all other viral subpopulations were non-significant. Significance was seen in C values of 0.87, whilst C values of 0.98–1.02 showed non-significance. At St Kilda, all of the viral subpopulations had non-significant Geary’s C values. C values were higher than what was seen in the bacteria at St Kilda, being between 0.96 and 1.02, which was similar to the C values obtained for the viruses at Noarlunga (Table S3 and S4).

Using Geary’s C to look in more detail at the randomly distributed subpopulations that showed positive to negative spatial autocorrelation within the Moran correlograms revealed they had significant Geary’s C values (Table S3 and S4). These subpopulations were the bacterial subpopulations in microplate 1, as well as the HDNA 1 subpopulation in microplate 2 at St Kilda and the bacterial subpopulations and VLP 2 and total virus populations in microplate 1 at Noarlunga.

## Discussion

### Bacterioplankton and virioplankton distributions

#### Surface gradients

Strong surface gradients were found at St Kilda, which are well established along with characteristic low dissolved oxygen levels [35]. These strong gradients rising from the sediment surface showed up to a 15-fold change in abundance over the 11.3 cm vertical sampling distance collected above the sediment-water interface, The presence of these gradients is perhaps due to the strong input of organic matter from the mangroves [46]. In support of this, Seymour *et al.* [35] found microscale microbial distributions were highly correlated to nutrient concentration gradients. Seymour *et al.* [15] found the shear velocities at St Kilda at the time of sampling were not high enough for the resuspension of microbes. Given the protected nature of the environment, resuspension may be minimal compared to exposed sites and would not play a role in the surface gradients observed.

Bacteria and viruses immediately above the sediment are exposed to a variety of biological and non-biological parameters that will affect their distribution [35]. At both sampling sites clear abundance gradients were often seen in relation to distance from the sediment-water interface. The remineralisation of organic nutrients is known to cause gradients with dissolved and particulate matter, which usually show decreasing concentrations as the distance from the sediment-water surface increases [35], [59].

#### Hotspots and coldspots

Previous studies could not determine whether a high or low point in a one dimensional profile was a gradient, a multiple point hotspot/coldspot or a single point maximum/minimum [35]. In this study, two-dimensional sampling revealed the presence of single and multiple point hotspots and single point coldspots in bacterial and viral populations at Noarlunga and St Kilda. In previous studies, local increases in abundance of up to 2 times above background over a distance of 1 cm have been considered hotspots [13], [14], [35], [43], whereas this study showed hotspots with increases of up to 2585 and 45.2 times cm^−1^ in viral and bacterial subpopulations above the sediment-water interface. These hotspots were high in bacteria and viruses, consistent with bacteriophage infection being dependent on host density [15]. The correlation of these bacterial and viral hotspots suggests a repeated viral infection and lysis cycle for at least some of the bacterial species present [12], [13], [15], which, in turn, suggests that groups of picoplankton, at least close to the sediment interface remain close together for extended periods. Through entrainment in the overlying water these near-sediment hotspots may act to inoculate the overlying water column. With viral hotspots showing increases of up to 2585 times cm^−1^ this would contribute substantially to the abundance of viral particles hence playing a role in viral production and succession. In the case of coldspots, correlations were seen with viral subpopulations but not with bacterial subpopulations. There are many possible dynamics at play here. For instance, this may indicate these coldspots are capturing the stable bacterial community numbers, which have not yet been exposed to viral communities or perhaps areas in which compatible viral particles were not present hence leading to viral decay or extensive grazing as the viruses would not have been able to protect themselves from external sources by infecting the bacteria present. Another possible explanation for these coldspots in viruses could be that they are microenvironments that favour lysogeny over lysis, such as areas of low nutrient concentration [60]. The wide range of hotspot values (Figure. S4) suggests that all of these and possibly more mechanisms are generating hotspots. Of course, the actual processes occurring in these hotspots and coldspots cannot be determined with biomass values alone and call for microscale nutrient and species analysis.

At St Kilda, patchy viral and bacterial hotspots and coldspots were identified and, in the case of the hotspots, often coincided. These hotspots and coldspots resulted in changes in spatial heterogeneity of 10.5-fold cm^−1^ for viruses and 45-fold cm^−1^ for bacteria. These results are much higher than the previously found 1.4- and 1.5-fold cm^−1^ increases found in total virus and total bacteria by Seymour *et al.* [35]. As these hotspots and coldspots often occurred within single sampling points of 0.9 cm distance this highlights the importance of high sampling resolution and 2-dimensional arrays when studying the distributions of microbial communities.

The null hypothesis tested in this study was that patchiness has no effect on mean biomass estimates and therefore the mean field approach is an accurate estimate of biomass. This null hypothesis was rejected with the presence of patchy hotspots and coldspots in this study showing differences of up to four orders of magnitude in biomass. In the most extreme case, if the mean field approach had been employed without sufficient replication, estimates in microbial biomass would be off by a factor of 1,560.

Previous studies have also lacked a definition for hotspots and coldspots making comparative analysis impossible. In this study rank abundance graphs have been used as a method for determining what sample values are hotspots, coldspots or background. Background values fitted a linear trend as these values are random and are an indicator of equilibrated additive and reductive processes. Such additive processes in bacterial and viral communities could be reproduction or aggregation, whilst reductive processes could relate to grazing, decay or lysis events.

Hotspots were identified as the sample points that exceeded the linear fit and exhibited a steep power law trend, indicating their non-random nature whilst the coldspots were characterised by a linear trend with slope values much higher than those of the background values due to large differences seen between adjacent value points on the graph (Figure. 4A–D). The hotspots indicate a favouring towards additive processes and coldspots indicate a favouring towards reductive processes resulting in a non-equilibrated state when compared to the background values. Exponent values for the hotspots were similar at Noarlunga and St Kilda and ranged from −0.1 to −1.4 suggesting this is the level of structure seen within bacterial and viral communities. Exponent values at and below −1 indicated considerable self-organisation and strong patches [61].

The background values fit single or multiple linear trends with St Kilda being characterised primarily by single linear trends whilst Noarlunga was characterised primarily by two linear trends within its background values for bacterial and viral communities. This could be an indication of different randomisation processes present. For instance, when considering bacteria, the presence of two linear trends seen at Noarlunga could indicate equilibrium between grazing and aggregation in one linear trend whilst the second may indicate equilibrium between reproduction and lysis. By using these rank abundance graphs, not only are hotspots and coldspots identified but patterns within the background values are able to be observed. Experimental work will be required to distinguish between randomisation processes.

### Bacterial and viral abundances

The LDNA and HDNA bacterial subpopulations observed have been described previously as different phylogenetic groups present within the environment [62] or as bacteria with differing activity levels [64]–[66]. In the former context, the total mean abundance of HDNA 2 at St Kilda and HDNA 1 at Noarlunga being higher than the total mean abundance of the other LDNA and HDNA subpopulations, may be explained by the species within these subpopulations being more efficient at utilising resources or avoiding predatory attack [62]. The total mean abundances also indicated HDNA 1 at St Kilda and LDNA at Noarlunga had the lowest abundances of the bacterial subpopulations which could be due to the species within HDNA 1 at St Kilda and LDNA at Noarlunga being more susceptible to infection or grazing [35], [62], [63]. In addition, higher concentrations of predators are found within the first few centimetres above the sediment-water interface, such as heterotrophic nanoflagellates [35]. However, in the context of differing bacterial activity, where the LDNA subpopulation represents dormant bacterial cells and the HDNA subpopulations represent active cells, the higher abundances of the HDNA subpopulations seen at both sites when compared to the LDNA subpopulation would be explained by the ability of the HDNA subpopulations to replicate and grow, hence leading to their higher abundances [35], [64]–[66]. In the context of different species, the hotspots would represent accumulation of chemotactic bacteria and the coldspots areas they had left.

Another factor controlling the abundances of the bacterial subpopulations is the viral populations present. The total mean abundance of the VLP 1 subpopulation was larger than the total mean abundance of the VLP 2 subpopulation at both sites. This has been found in previous studies, with aquatic samples being dominated by the viral population that contains small viruses between 30 and 60 nm, which corresponds to the VLP 1 subpopulation within the cytograms (Figure.1A and C) [48], [67].

### Bacterial and viral coupling

Seymour *et al.* [35] did not find a correlation between total bacteria and viruses at this scale. In this study, a significant relation existed between total bacteria and total viruses at Noarlunga, whilst at St Kilda, a relation did not exist (Figure. 2A and B). This may be due to the presence of different microbial communities and the difference in environmental parameters between the two sampling sites. The dynamic nature of the environments may have led to short-lived events, such as nutrient patches, causing the aggregation and dispersion of bacteria over the course of minutes to hours. This initial increase in bacterial abundance would lead to increased viral production via lysis hence decoupling the relationship between viral and bacterial populations which may be why no relationship was seen between bacteria and viruses at St Kilda [15], [38]. However, stages in between the bacterial aggregation and bacterial death via viral lysis may have been captured at Noarlunga and would explain the correlation between the bacteria and viruses in this environment.

While Seymour *et al.* [35] found no correlation between total bacteria and viruses; correlations were seen between the VLP 1, VLP 2 and LDNA subpopulations and the VLP 2 and HDNA 2 subpopulations. Therefore, showing subpopulations have independent and potentially more complex associations than what is seen in total bacteria and virus populations. These results imply that total populations reflect system biomass and the processes that may impact on this, such as grazing; whilst single subpopulations reflect the phylogenetic changes in the community from such processes as phage infection and lysis. As the individual subpopulations can be decoupled, with phylogenetic fluctuations being independent of the total system biomass, this explains why correlations were seen in the totals but not in the individual subpopulations. While the correlations are not mechanisms, they show that possible relationships exist that could be further investigated. These statements are based on biomass only. Taxonomic identification of the community will be part of a manuscript in preparation.

### Spatial autocorrelation

#### Non-random vs random distribution

At St Kilda, non-random distributions were seen in the LDNA subpopulation of microplate 2 and 3 and the HDNA 2 subpopulation and total bacteria of microplate 2 due to these subpopulations having significant Moran’s I values and z scores larger than the +1.96 cut-off value. Whilst all viral subpopulations were randomly distributed due to having non-significant Moran’s I values. This may be due to motile bacterial subpopulations congregating around nutrient gradients and patches or resuspension of bacteria from the sediment leading to the observed surface gradients [68]. These non-random distributions may not have been seen in the viral subpopulations as viruses are not motile and are unable to respond to chemical signals.

The significant Moran’s I values seen in the bacterial subpopulations at St Kilda were positive, indicating clustering and spatial dependence. This clustering indicates that high values are located close together and low values are located close together, indicating the presence of ‘hotspots’ and ‘coldspots’ or gradients of high or low values. Moran’s I values ranged from 0.04 to 0.07 for clustered distributions. These values are low when considering a Moran’s I value of +1 indicates perfect clustering. These values are also low compared to Waters *et al.* [17] which had significant Moran’s I values between 0.082–0.180. These low values may indicate a low level of clustering present within these distributions.

At Noarlunga, all the bacterial and viral subpopulations were randomly distributed due to having non-significant Moran’s I values. This indicates that no spatial autocorrelation was present amongst the bacterial and viral subpopulation distributions and that there is no spatial dependency within the distributions of the bacterial or viral subpopulations locally or globally. On a large scale this implies that environmental processes, such as turbulence, randomize the spatial distribution of bacterial and viral subpopulations at Noarlunga.

The non-random and random bacterial distributions found at St Kilda and Noarlunga coincide with the findings of Waters *et al.* [17], which found higher spatial complexity within low-energy, high-chlorophyll environments such as St Kilda mangroves than in high-energy, low-chlorophyll environments such as Noarlunga. As St Kilda mangroves is a low-energy system with high productivity, due to the presence of dense microbial mats and mangrove forests causing stagnation in the lagoons, this may allow the development and maintenance of nutrient surface gradients [12], [13], [35], [46].

#### Correlogram autocorrelation patterns

At St Kilda, the positive spatial autocorrelation seen within 5.9 cm of the sediment-water interface in most bacterial subpopulations indicates the presence of potential nutrient gradients that lead to bacterial clustering and the formation of surface gradients. The negative spatial autocorrelation seen beyond 5.9 cm from the sediment-water interface shows the area at which such gradients dissipate, leading to dispersed bacterial distributions. This trend may be due to eddies, Nyquist frequency limitation or a nepheloid layer. The presence of eddies could cause resuspension of particles via eddy penetration leading to a short-time mixing effect and turbulence [69]–[71]. Whereas, Nyquist frequency, whereby sampling frequency should be at least twice the highest frequency present in the sample data, may have contributed to this trend as if the sampling frequency was insufficient this would lead to the presence of aliases and distortions in the dataset [72]. The presence of a 5.9 cm nepheloid layer would result in high levels of suspended particulate matter being present at sediment-water interface and the presence of such layer may largely depend on the time of sampling as nepheloid layers are subject to change depths depending on the season [73], [74].

Taking into account the study by Sokal [57] which showed that high Moran’s I values not accompanied by high Geary’s C values are representative of deterministic patterning of extreme values and the reverse implies that extreme values are randomly distributed and are hence spatially independent. Then for the bacterial subpopulations which showed positive spatial autocorrelation 5.9 cm from the sediment-water interface and then negative spatial autocorrelation beyond 5.9 cm, with relatively low corresponding Geary’s C values between 0.73 and 0.83 suggests there was deterministic patterning of extreme values. Whereas, the positive to negative spatial autocorrelation trend within the Moran correlograms of the bacterial subpopulations that were found to be randomly distributed and had low Moran’s I values which were less than the estimated cut-off value for clustered distributions but also had relatively low Geary’s C values between 0.85 and 0.89 may indicate that globally these populations are spatially independent and hence randomly distributed, which is why the Moran’s *I* value is low, but locally they show non-random distributions. In addition, the LDNA bacterial subpopulation at St Kilda in microplate 3 showed non-random distributions in the Moran’s I testing however it had a higher Geary’s C value than the other non-randomly distributed populations (0.92 compared to 0.73–0.83) which was more similar to the Geary’s C values of the randomly distributed subpopulations that showed local association of 0.85–0.89. According to Sokal [57] this suggests that this subpopulation may be randomly distributed as the non-random result from the Moran’s I was skewed by extreme values within the data set. This again highlights the importance of using Moran’s I in conjunction with Geary’s C as globally bacterial subpopulations may be spatially independent however locally they may be spatially dependent. As Geary’s C is able to account for extreme outliers that may skew the overall result it is important to use in microbial distribution studies which have shown increases of up to 45 and 2500 times cm^−1^ in bacteria and viruses.

### ‘Mean field’ approach limitations

The microscale spatial heterogeneity of bacterial and viral populations in this study would be underestimated, by more than 3 orders of magnitude in some cases, had the mean field approach been employed [75]–[77]. This is due to the presence of single point hotspots and coldspots containing more than 30% and less than 0.0008% of the total biomass within each microplate. As the distributions were random at Noarlunga but non-random at St Kilda the mean would not be representative of microbial biomass.

## Conclusion

Spatial heterogeneity in microbial abundance has been found within the 11.3 cm directly above the sediment-water interface at Noarlunga estuary and St Kilda mangroves. This heterogeneity was present in the form of single and multiple point hotspots at Noarlunga and hotspots and surface gradients at St Kilda which resulted in high levels of viral and bacterial abundance being found near the sediment-water interface. These patterns in microbial distributions have been seen in previous studies but to a lesser extent. At St Kilda, gradients in bacterial and viral abundance were much higher than previously reported with a 45- and 10.5-fold change cm^−1^ found compared to the previously reported 1.5-fold change in abundance cm^−1^ in bacteria [35] and 1.4-fold change cm^−1^ in viruses [15], [35]. At Noarlunga, bacterial and viral abundance showed a 12.9– and 2584-fold change cm^−1^. This large fold-change found cm^−1^ would have been missed if lower resolution sampling was used. This high level of microscale heterogeneity highlights the importance of analysing microbial spatial distribution on the level of what individual cells experience rather than using a ‘mean field’ approach. In addition, this high level of microscale heterogeneity also illustrates the necessity and applicability of the novel two-dimensional high resolution sampler used in this study which was capable of collecting eight vertical profiles. The use of this sampler allowed the discrimination of single and multiple point hotspots and/or surface gradients, which was not possible in past studies as one-dimensional samplers were used.

Bacterial subpopulations were found to be randomly distributed at Noarlunga and non-randomly distributed at St Kilda with reasons for this finding relating to the low energy-nature of St Kilda allowing the formation of sediment-water interface nutrient and microbial surface gradients. Viral subpopulations only showed random distributions at both sites. Moran’s *I* and Geary’s C reveal that spatial autocorrelation amongst bacterial subpopulations at St Kilda show a positive to negative trend 5.9 cm from the sediment-water interface, indicating a mechanism, whether this be sampling alias or particulate resuspension, is present leading to this clustering and dispersal of bacterial populations.

Despite hotspots being ubiquitous features of microbial distributions, past studies lacked a universal definition. They qualitatively classified patches as regions of high microbial abundance that ‘exceed’ or are ‘elevated above’ background values [12], [13], [43]. This paper provides a new quantitative method for determining hotspots, coldspots and background values within microscale microbial distributions. It is hoped that this method will be used in future microbial microscale distributions to allow comparative analyses among studies.

## Supporting Information

Figure S1 **Collection of Vertical Profiles.** The use of an 8×12 96-well microplate allowed the two-dimensional array of 8 vertical profiles (row A, B, C, D, E, F, G and H) per microplate which consisted of 12 sampling wells per vertical profile.
(TIF)

Figure S2 **Comparisons of bacterial and viral subpopulations and viral subpopulations at Noarlunga via vertical depth profiles.** Bacterial and viral subpopulations of all three microplates (n = 270). **A** Total mean LDNA, HDNA 1 and HDNA 2; **B** Total mean VLP 1 and VLP 2. Error bars represent the 95% confidence intervals obtained from each subpopulation of all three replicates (n = 12).
(TIF)

Figure S3 **Mean vertical profile of VLP 1 and LDNA, microplate one at Noarlunga (n = 90).** Error bars represent the 95% confidence intervals obtained from one replicate (n = 12).
(TIF)

Figure S4 **Adjacent orthogonal gradient distributions of bacteria and viruses as rank abundance. St Kilda: A** VLP 1, **B** HDNA 2. **Noarlunga:** **C** VLP 2, **D** HDNA 2. Each distribution is ordered as a rank abundance. In each case the first rank was used for the 2 dimensional plots. A logarithmic trend-line was the best fit for each distribution, with the equations and r^2^ for **A** y = −4×10^6^ ln(x)+2×10^7^ (R^2^ = 0.97), **B** y = −7×10^4^ ln(x)+4×10^6^ (R^2^ = 0.99), **C** y = −2×10^6^ ln(x)+8×10^6^ (R^2^ = 0.98), **D** y = −8×10^4^ ln(x)+4×10^5^ (R^2^ = 0.92). The p values are <<0.05 in all cases.
(TIF)

Table S1 **Mean viral abundances per microplate at Noarlunga and St Kilda.**
(DOCX)

Table S2 **Mean bacterial abundances per microplate at Noarlunga and St Kilda.**
(DOCX)

Table S3 **Comparison of the Moran’s I values and Geary’s C values obtained for each bacterial subpopulation at Noarlunga and St Kilda.**
(DOCX)

Table S4 **Comparison of the Moran’s I values and Geary’s C values obtained for each viral subpopulation at Noarlunga and St Kilda.**
(DOCX)
