# Supplementary figures and images for: Virio- and Bacterioplankton Microscale Distributions at the Sediment-Water Interface
Source: PLoS One. 2014 Jul 24;9(7):e102805. doi: 10.1371/journal.pone.0102805 (PMC4109957; doi:10.1371/journal.pone.0102805)

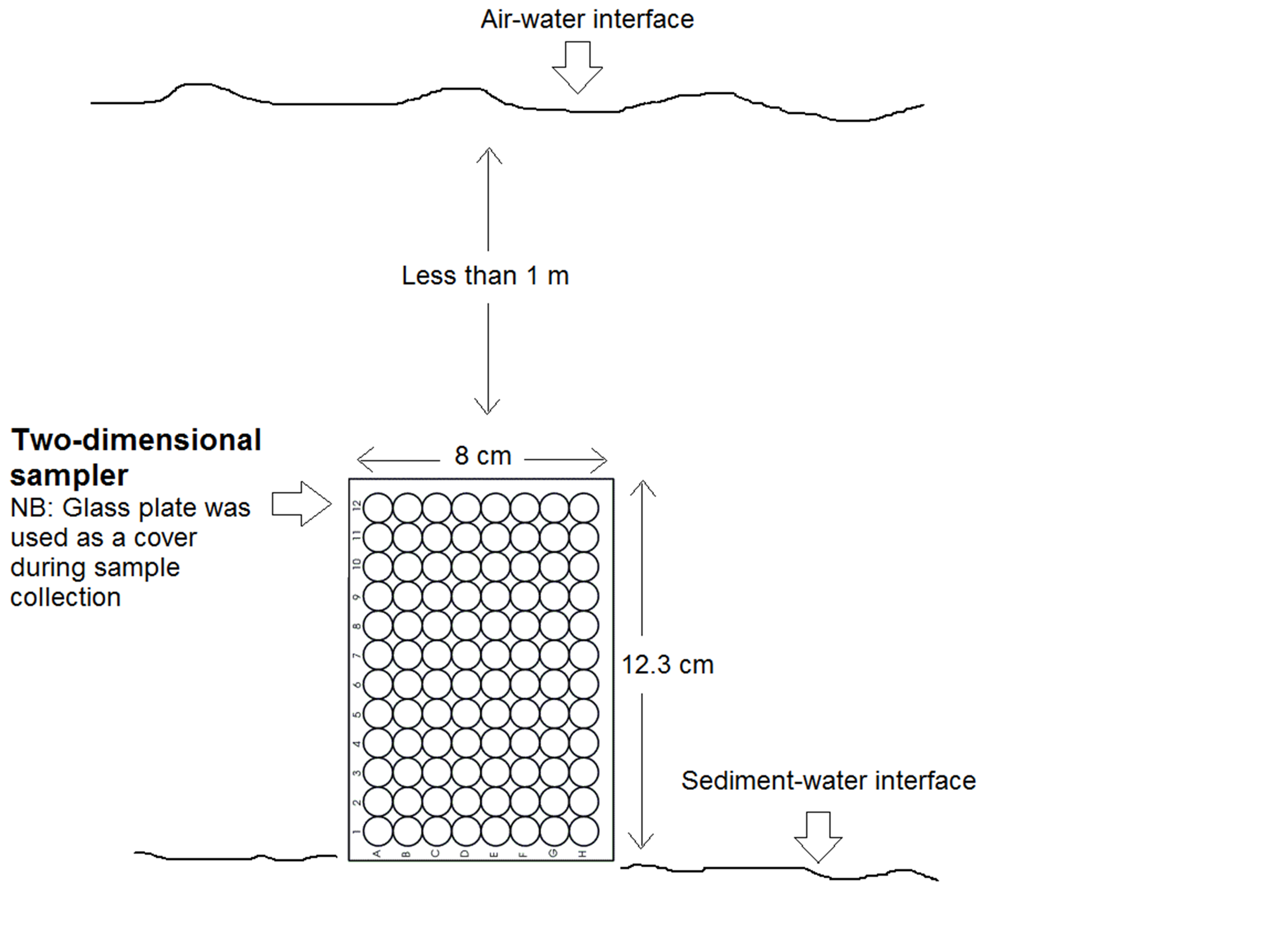

Supplement: Figure S1 — Collection of Vertical Profiles. The use of an 8×12 96-well microplate allowed the two-dimensional array of 8 vertical profiles (row A, B, C, D, E, F, G and H) per microplate which consisted of 12 sampling wells per vertical profile. (TIF) [file pone.0102805.s001.tif]

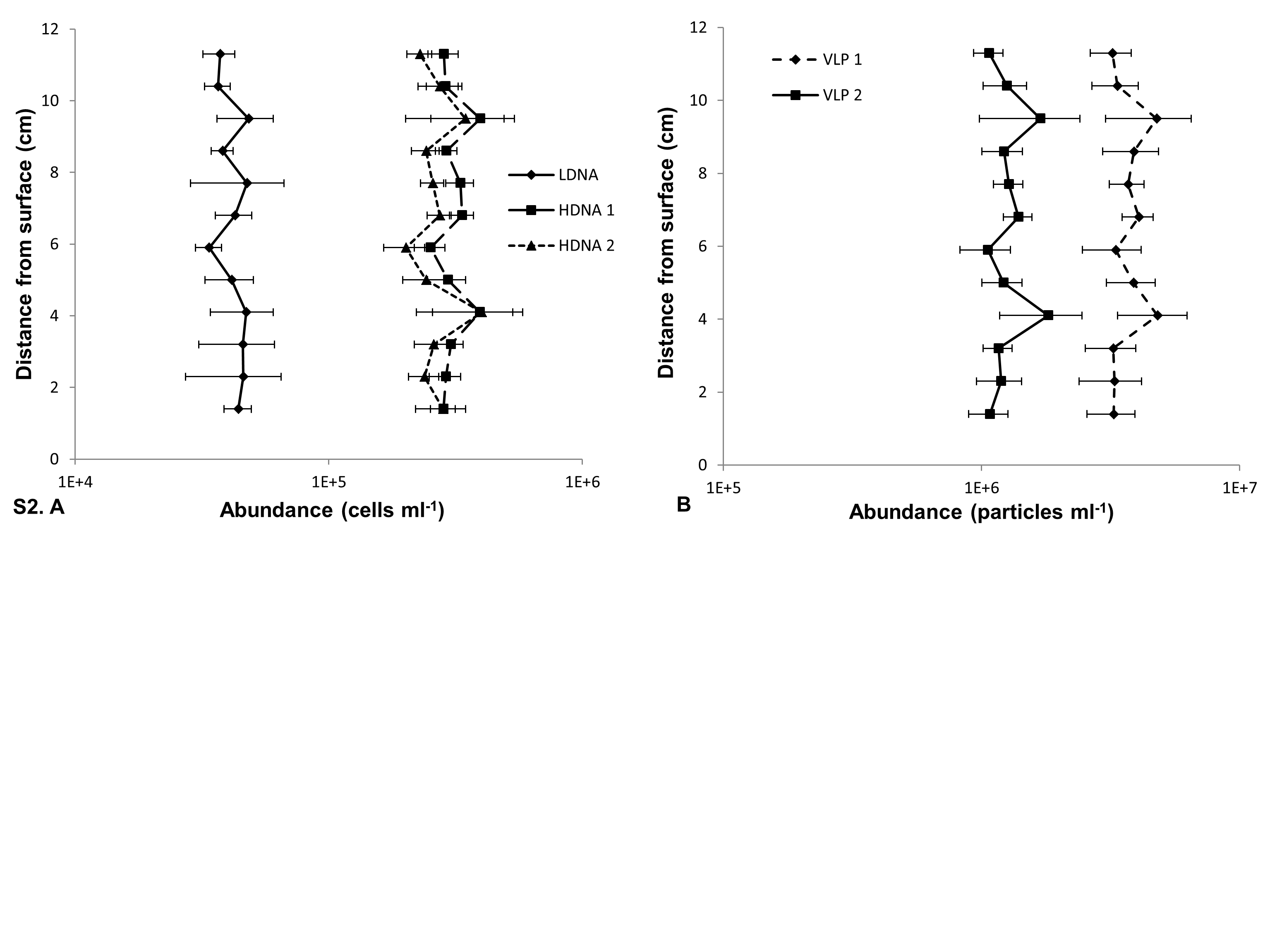

Supplement: Figure S2 — Comparisons of bacterial and viral subpopulations and viral subpopulations at Noarlunga via vertical depth profiles. Bacterial and viral subpopulations of all three microplates (n = 270). A Total mean LDNA, HDNA 1 and HDNA 2; B Total mean VLP 1 and VLP 2. Error bars represent the 95% confidence intervals obtained from each subpopulation of all three replicates (n = 12). (TIF) [file pone.0102805.s002.tif]

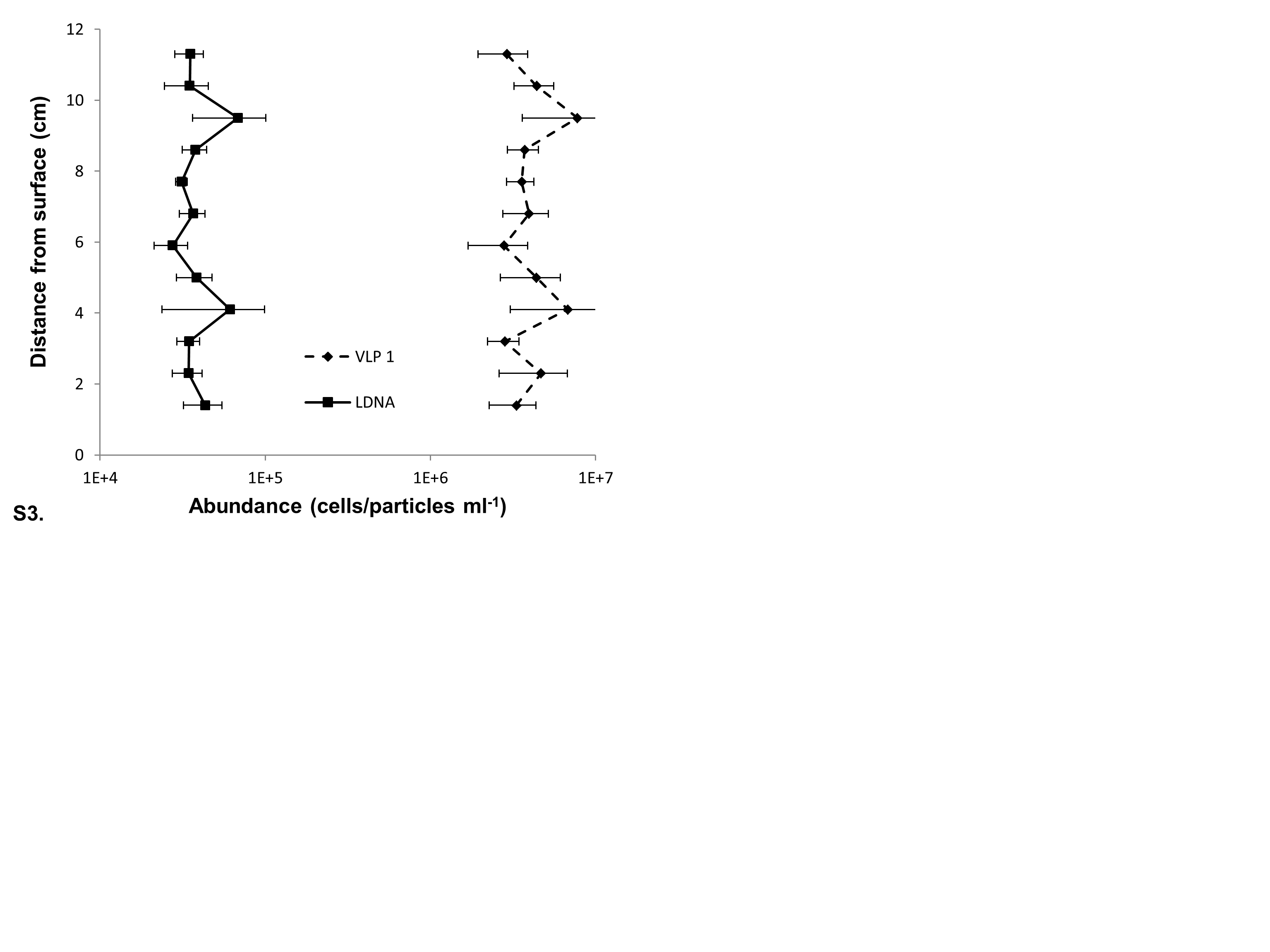

Supplement: Figure S3 — Mean vertical profile of VLP 1 and LDNA, microplate one at Noarlunga (n = 90). Error bars represent the 95% confidence intervals obtained from one replicate (n = 12). (TIF) [file pone.0102805.s003.tif]

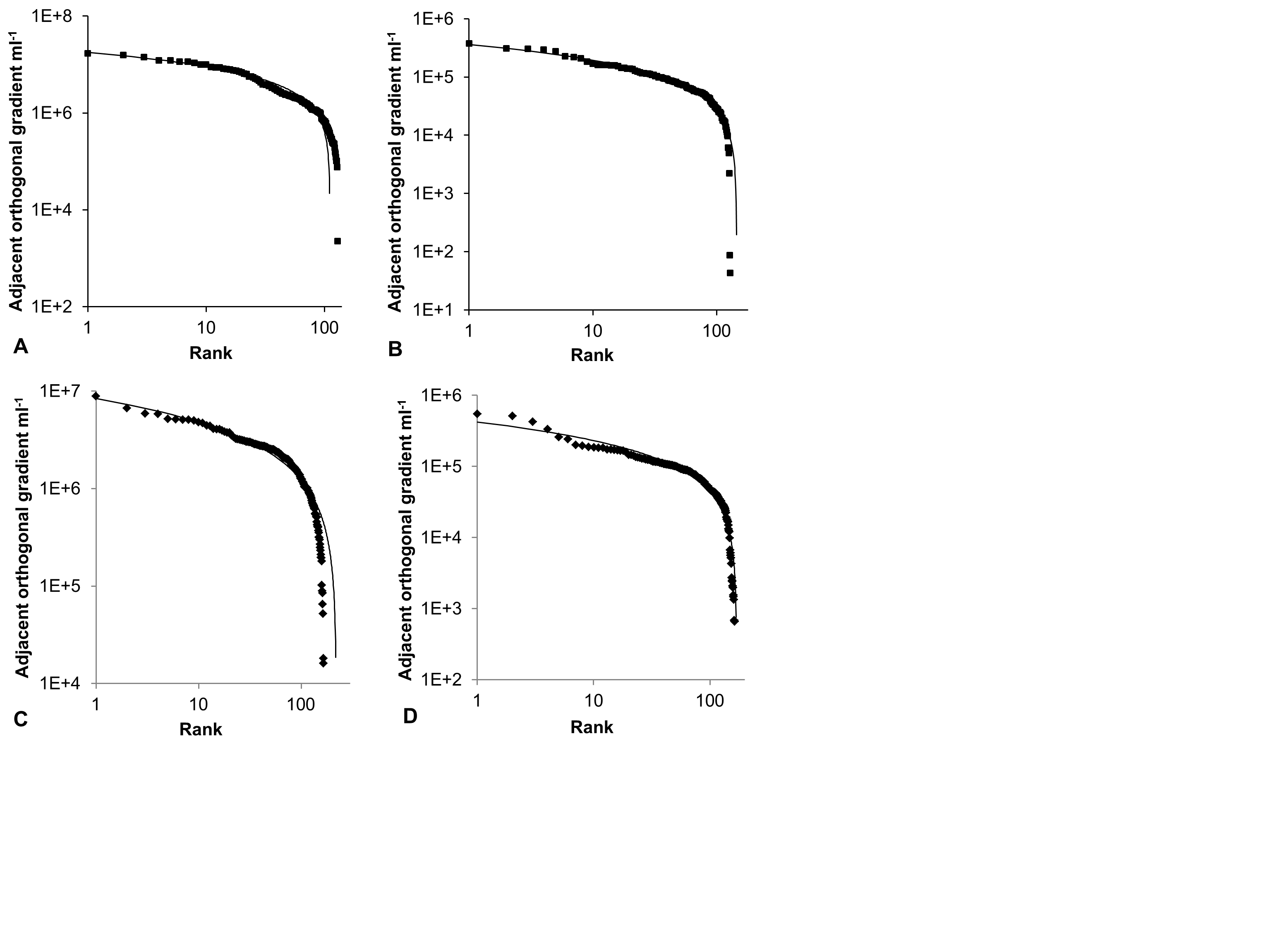

Supplement: Figure S4 — Adjacent orthogonal gradient distributions of bacteria and viruses as rank abundance. St Kilda: A VLP 1, B HDNA 2. Noarlunga: C VLP 2, D HDNA 2. Each distribution is ordered as a rank abundance. In each case the first rank was used for the 2 dimensional plots. A logarithmic trend-line was the best fit for each distribution, with the equations and r2 for A y = −4×10^6^ ln(x)+2×10^7^ (R2 = 0.97), B y = −7×10^4^ ln(x)+4×10^6^ (R2 = 0.99), C y = −2×10^6^ ln(x)+8×10^6^ (R2 = 0.98), D y = −8×10^4^ ln(x)+4×10^5^ (R2 = 0.92). The p values are <<0.05 in all cases. (TIF) [file pone.0102805.s004.tif]
