# Supplementary material for: Virio- and Bacterioplankton Microscale Distributions at the Sediment-Water Interface
Source: PLoS One. 2014 Jul 24;9(7):e102805. doi: 10.1371/journal.pone.0102805 (PMC4109957; doi:10.1371/journal.pone.0102805)
Supplement: Table S1 — Mean viral abundances per microplate at Noarlunga and St Kilda. (DOCX) [file pone.0102805.s005.docx]

**Table S1.**

| **Population** | **Microplate Number** | **Viral Abundance**  x 10^6^ particles ml^-1^ (95%CI, n) | |
| --- | --- | --- | --- |
|  |  | **Noarlunga** | **St Kilda** |
| VLP1 | 1 | 4.3 (0.6, 89) | 8.4 (1.0, 69) |
|  | 2 | 4.0 (0.37, 94) | 6.6 (0.7, 58) |
|  | 3 | 2.9 (0.35, 86) | 7.2 (0.8, 82) |
| VLP2 | 1 | 1.5 (0.3, 89) | 0.7 (0.1, 69) |
|  | 2 | 1.3 (0.1, 94) | 0.6 (0.07, 58) |
|  | 3 | 1.0 (0.1, 86) | 0.5 (0.05, 82) |
| **Total Virus** | 1 | 5.75 (0.9, 89) | 9.15 (1.1, 69) |
|  | 2 | 5.3 (0.4, 94) | 7.25 (0.75, 58) |
|  | 3 | 3.9 (0.4, 86) | 7.8 (0.8, 82) |
