# Supplementary material for: Virio- and Bacterioplankton Microscale Distributions at the Sediment-Water Interface
Source: PLoS One. 2014 Jul 24;9(7):e102805. doi: 10.1371/journal.pone.0102805 (PMC4109957; doi:10.1371/journal.pone.0102805)
Supplement: Table S2 — Mean bacterial abundances per microplate at Noarlunga and St Kilda. (DOCX) [file pone.0102805.s006.docx]

**Table S2.**

| **Population** | **Microplate Number** | **Bacterial Abundance**  x 10^5^ cells ml^-1^ (95%CI, n) | |
| --- | --- | --- | --- |
|  |  | **Noarlunga** | **St Kilda** |
| LDNA | 1 | 0.4 (0.05, 89) | 3.4 (0.65, 69) |
|  | 2 | 0.4 (0.05, 94) | 3.3 (0.8, 58) |
|  | 3 | 0.5 (0.1, 86) | 2.4 (0.1, 82) |
| HDNA 1 | 1 | 3.6 (0.5, 89) | 2.4 (0.9, 69) |
|  | 2 | 2.9 (0.2, 94) | 2.8 (1.3, 58) |
|  | 3 | 2.9 (0.2, 86) | 1.4 (0.1, 82) |
| HDNA 2 | 1 | 3.2 (0.6, 89) | 6.8 (4.1, 69) |
|  | 2 | 2.8 (0.2, 94) | 8.1 (3.7, 58) |
|  | 3 | 2.1 (0.2, 86) | 3.5 (0.2, 82) |
| **Total Bacteria** | 1 | 7.25 (1.15, 89) | 9.8 (1.4, 68) |
|  | 2 | 6.05 (0.3, 94) | 14.1 (5.6, 58) |
|  | 3 | 5.5 (0.35, 86) | 7.4 (0.3, 82) |
