# Supplementary material for: Virio- and Bacterioplankton Microscale Distributions at the Sediment-Water Interface
Source: PLoS One. 2014 Jul 24;9(7):e102805. doi: 10.1371/journal.pone.0102805 (PMC4109957; doi:10.1371/journal.pone.0102805)
Supplement: Table S3 — Comparison of the Moran’s I values and Geary’s C values obtained for each bacterial subpopulation at Noarlunga and St Kilda. (DOCX) [file pone.0102805.s007.docx]

**Table S3.**

| **Microplate Number** | **Population** | **Noarlunga** | | **St Kilda** | |
| --- | --- | --- | --- | --- | --- |
|  |  | **Moran’s *I***  **(p-value)** | **Geary’s C**  **(p-value)** | **Moran’s *I***  **(p-value)** | **Geary’s C**  **(p-value)** |
| 1 | LDNA | -0.008 (n.s) | 0.87 (0.0001) | 0.021(n.s) | 0.85 (0.0001) |
|  | HDNA 1 | -0.008 (n.s) | 0.87 (0.0001) | 0.004 (n.s) | 0.86 (0.0001) |
|  | HDNA 2 | -0.008 (n.s) | 0.87 (0.0001) | 0.006 (n.s) | 0.85 (0.0001) |
|  | **Total Bacteria** | -0.008 (n.s) | 0.87 (0.0001) | 0.019 (n.s) | 0.89 (0.001) |
| 2 | LDNA | -0.017 (n.s) | 0.96 (n.s) | 0.062 (0.001) | 0.73 (0.0001) |
|  | HDNA 1 | -0.004 (n.s) | 0.98 (n.s) | 0.036 (n.s) | 0.75 (0.0001) |
|  | HDNA 2 | -0.004 (n.s) | 0.98 (n.s) | 0.070 (0.001) | 0.83 (0.0001) |
|  | **Total Bacteria** | 0.007 (n.s) | 0.94 (n.s) | 0.070 (0.001) | 0.77 (0.0001) |
| 3 | LDNA | -0.017 (n.s) | 0.98 (n.s) | 0.039 (0.01) | 0.92 (0.001) |
|  | HDNA 1 | -0.011 (n.s) | 0.99 (n.s) | 0.0002 (n.s) | 0.99 (n.s) |
|  | HDNA 2 | -0.009 (n.s) | 1.01 (n.s) | 0.002 (n.s) | 0.97 (n.s) |
|  | **Total Bacteria** | -0.012 (n.s) | 0.98 (n.s) | 0.007 (n.s) | 0.96 (n.s) |
