# Supplementary material for: Virio- and Bacterioplankton Microscale Distributions at the Sediment-Water Interface
Source: PLoS One. 2014 Jul 24;9(7):e102805. doi: 10.1371/journal.pone.0102805 (PMC4109957; doi:10.1371/journal.pone.0102805)
Supplement: Table S4 — Comparison of the Moran’s I values and Geary’s C values obtained for each viral subpopulation at Noarlunga and St Kilda. (DOCX) [file pone.0102805.s008.docx]

**Table S4.**

| **Microplate Number** | **Population** | **Noarlunga** | | **St Kilda** | |
| --- | --- | --- | --- | --- | --- |
|  |  | **Moran’s *I***  **(p-value)** | **Geary’s C**  **(p-value)** | **Moran’s *I***  **(p-value)** | **Geary’s C**  **(p-value)** |
| 1 | VLP1 | -0.001 (n.s) | 1.02 (n.s) | -0.026 (n.s) | 1.01 (n.s) |
|  | VLP2 | -0.008 (n.s) | 0.87 (0.0001) | -0.023 (n.s) | 0.96 (n.s) |
|  | **Total Virus** | -0.008 (n.s) | 0.87 (0.0001) | -0.021(n.s) | 1.00 (n.s) |
| 2 | VLP1 | 0.007 (n.s) | 1.01 (n.s) | -0.044 (n.s) | 1.02 (n.s) |
|  | VLP2 | 0.004 (n.s) | 0.99 (n.s) | -0.029 (n.s) | 0.97 (n.s) |
|  | **Total Virus** | 0.009 (n.s) | 1.01 (n.s) | -0.044 (n.s) | 1.00 (n.s) |
| 3 | VLP1 | 0.014 (n.s) | 1.00 (n.s) | 0.015 (n.s) | 0.96 (n.s) |
|  | VLP2 | 0.003 (n.s) | 0.98 (n.s) | 0.0007 (n.s) | 1.00 (n.s) |
|  | **Total Virus** | 0.013 (n.s) | 0.99 (n.s) | 0.018 (n.s) | 0.96 (n.s) |
